# Additional benefit of standardized computed tomography-based lymph node assessment utilizing Node-RADS in esophageal adenocarcinoma

**DOI:** 10.1016/j.esmogo.2026.100321

**Published:** 2026-03-26

**Authors:** J.J. Staudacher, C. Treese, E.M. Köhne, P. Jäger, I. Pozios, F. Branchi, B. Siegmund, S. Daum, L.-A. Schaafs, F. Elsholtz

**Affiliations:** 1 Department of Gastroenterology, Infectious Diseases and Rheumatology, Charité—Universitätsmedizin Berlin, Corporate Member of Freie Universität Berlin and Humboldt-Universität zu Berlin, Campus Benjamin Franklin, Berlin, Germany; 2 Berlin Institute of Health at Charité, Berlin, Germany; 3 Department of General and Visceral Surgery, Charité—Universitätsmedizin Berlin, Corporate Member of Freie Universität Berlin and Humboldt-Universität zu Berlin, Campus Benjamin Franklin, Berlin, Germany; 4 Cluster of Excellence ImmunoPreCept, Charité—Universitätsmedizin Berlin, Berlin, Germany; 5 Department of Radiology, Charité—Universitätsmedizin Berlin, Corporate Member of Freie Universität Berlin and Humboldt-Universität zu Berlin, Campus Benjamin Franklin, Berlin, Germany

**Keywords:** esophageal adenocarcinomas, staging, lymph node metastasis, Node-RADS, computed tomography, endoscopic ultrasound

## Abstract

**Background:**

Accurate lymph node staging is crucial for treatment stratification in esophageal adenocarcinoma. While endoscopic ultrasound (EUS) is standard for locoregional staging, computed tomography (CT) remains essential for detecting distant metastasis. This study aims to evaluate the Node Reporting and Data System (Node-RADS) framework, developed to standardize CT-based lymph node evaluation, in locally advanced esophageal adenocarcinomas.

**Materials and methods:**

We retrospectively included patients with histologically confirmed esophageal adenocarcinoma who were treated with multimodal therapy and curative-intent surgery between 2017 and 2023. Preoperative CT scans were scored according to Node-RADS by two independent radiologists. Kaplan–Meier and log-rank tests assessed survival differences, and multivariable Cox regression was employed to identify independent prognostic factors.

**Results:**

Based on survival trajectory analysis, a score of ≥2 was defined as Node-RADS positive (nN+).This identified 84 nN+ and 75 nN− patients. Node-RADS positivity strongly correlated with EUS nodal status (*P* < 0.001, ϕ = 0.36) and pathological nodal involvement post-neoadjuvant therapy (*P* < 0.001, ϕ = 0.34). Patients with nN+ disease had shorter overall survival (46.2 versus 65.4 months, *P* < 0.001). Discordant cases between EUS and Node-RADS showed intermediate survival. In multivariable analysis, only nN status and nodal status by EUS independently predicted survival.

**Conclusions:**

Node-RADS provides a reproducible CT-based method for lymph node assessment in esophageal adenocarcinoma. It positively correlates with EUS and pathological nodal involvement and independently predicts survival. Node-RADS may complement EUS for staging, but prospective studies are needed to define optimal cut-offs and validate its clinical utility.

## Highlights

• Node-RADS provides a standardized CT-based framework for oncologic lymph node assessment.
• In esophageal adenocarcinomas, Node-RADS correlates strongly with EUS and pathological nodal status.
• Node-RADS positivity independently predicts shorter overall survival in these patients.
• Discordant Node-RADS and EUS nodal staging identify patients with intermediate survival.

## Introduction

Esophageal adenocarcinomas are aggressive tumors with rising incidence, particularly in Western countries.^1^ Early stages are managed by endoscopic or surgical resection, while locally advanced cases with nodal involvement or deep tumor infiltration receive perioperative treatment,^2^ first established in the FFCD^3^ and MAGIC trials. The current standard of care in locally advanced disease is FLOT chemotherapy, as defined by the ESOPEC trial.^4^ Recently, adding immune checkpoint inhibition to perioperative FLOT improved progression-free survival in the MATTERHORN trial likely setting a new standard of care.^5^

This multimodal approach has significantly improved survival compared with surgery alone but is associated with significant morbidity. In the AIO-FLOT-4 trial, only around half of patients completed the planned adjuvant part of FLOT therapy,^6^ and >70% of patients in the MATTERHORN trial experienced grade ≥3 adverse events.^5^ These findings highlight the burden of multimodal therapy and emphasize the need for accurate pretherapeutic staging to optimize treatment and avoid under- or overtreatment.

Lymph node staging is crucial for risk stratification and treatment planning. Current international guidelines, including those from the European Society for Medical Oncology (ESMO)^2^ and the National Comprehensive Cancer Network (NCCN),^7^ recommend comprehensive multimodal imaging for patients with potentially resectable esophageal carcinoma.

Endoscopic ultrasound (EUS) is the preferred method for locoregional staging of esophageal cancer, particularly for evaluating tumor depth (T stage) and regional lymph node involvement (N stage). Reported sensitivity for malignant lymph node detection ranges from 72% to 90%, with specificity between 65% and 85%, depending on operator skill and tumor location.^8^ However, stenotic tumors often limit adequate lymph node staging by EUS. Contrast-enhanced computed tomography (CT) remains the diagnostic standard for detection of distant metastasis, while playing a secondary role in the assessment of lymph node involvement.

A major limitation of CT-based lymph node staging is the absence of standardized malignancy criteria, leading to high interobserver variability and poor reproducibility across centers. To address this, the Node Reporting and Data System (Node-RADS) was developed to provide a standardized, reproducible framework for lymph node evaluation.^9^ Similar to BI-RADS for breast imaging^10^ or PI-RADS for prostate cancer,^11^ Node-RADS assigns scores from 1 (very low suspicion) to 5 (very high suspicion) based on morphologic features such as size and shape, thereby standardizing interpretation and increasing interobserver reliability.^9^

Despite its conceptual advantages, clinical application and validation of Node-RADS in specific tumor types are limited. Data on esophageal cancer—and particularly on esophageal adenocarcinomas—are sparse. A Chinese study reported high diagnostic accuracy in squamous-cell carcinoma,^12^ whereas a German retrospective study of preoperative CT scans in a mixed cohort of adenocarcinomas and squamous-cell carcinomas found only limited correlation between Node-RADS score and pathological findings.^13^

We hypothesized that Node-RADS provides added value over traditional EUS-based lymph node staging. To test this, we evaluated standardized CT-based lymph node staging following the Node-RADS approach retrospectively in a single-center cohort of locally advanced esophageal adenocarcinomas.

## Materials and methods

### Ethics approval

This study was conducted in accordance with all regulatory requirements and ethical guidelines for human research, including the Declaration of Helsinki and national data protection laws. Ethics approval was obtained before start of this analysis from the Institutional Review Board (IRB) of Charité—Universitätsmedizin Berlin (approval number: EA4/115/10).

### Patient selection

Patients were eligible for inclusion if they had a histologically confirmed diagnosis of esophageal adenocarcinoma, including tumors arising at the gastroesophageal junction classified as Siewert type I.^14^ Additional inclusion criteria were tumor staging carried out using EUS and contrast-enhanced CT scans with at least a portal venous phase, and treatment with multimodal therapy followed by curative-intent surgery between January 2017 and December 2023. Inclusion criteria required patients to be ≥18 years of age at diagnosis, and potential participants were identified via hospital electronic records using the International Classification of Diseases, 10th Revision (ICD-10) and OPS codes (Operationen- und Prozedurenschlüssel”; German procedure classification system); specifically, ICD-10 code C15.- and the OPS procedure codes 5-423, 5-424, 5-425, 5-426, and 5-429 including all subcodes were applied, respectively. The majority of patients (89.9%) received perioperative chemotherapy following the FLOT regimen, and the rest were treated with neoadjuvant radiochemotherapy following the CROSS regimen. No specific prehabilitation program was utilized, but all patients received psycho-oncologic support and nutritional support when indicated following current national guidelines.

### Data extraction and survival outcomes

For all included patients, demographic characteristics, tumor-specific variables, and treatment-related details were manually extracted from electronic health records by two independent investigators. Survival data were derived by cross-referencing patients with the German death registry. Overall survival (OS) was defined as the interval from initial histopathological diagnosis to death from any cause or date of cross-reference.

### Node-RADS and histopathological scoring

Initial staging CT scans at diagnosis were retrospectively assessed for lymph node involvement using Node-RADS,^9^ which scores nodes from 1 (very low likelihood) to 5 (very high likelihood of malignancy) based on size and morphology, with very large ‘bulk’ nodes automatically scored as 5. Two board-certified radiologists, each with >5 years of oncologic imaging experience, independently reviewed all scans in a blinded manner. CT examinations were evaluated which, due to the study setting, were carried out using various multislice CT scanners (64-320 rows) from different manufacturers. In order to achieve the greatest possible consistency in image quality, only examinations that included at least a portal venous phase (i.e. 70-90 s after contrast agent injection) were considered. Primary image acquisition was carried out with a slice thickness of 1.0 mm, and multiplanar reconstructions with soft tissue kernels were available in slice thicknesses between 1.0 and a maximum of 5.0 mm. The readers were allowed to choose freely between the available multiplanar reconstructions. Histopathological assessment was carried out on formalin-fixed, paraffin-embedded esophagectomy specimens obtained after neoadjuvant multimodal therapy. Surgical specimens were evaluated by board-certified pathologists according to standardized institutional protocols and the eighth edition of the Union for International Cancer Control (UICC)/American Joint Committee on Cancer (AJCC) TNM (tumor–node–metastasis) classification system for esophageal adenocarcinoma. Time from initial CT examination and histopathological examination differed interindividually, but was in the range of 10-15 weeks, also depending on treatment course (FLOT versus CROSS regimens). Becker score was assessed as described in the original publication.^15^

### Statistical analysis

All statistical analyses were carried out using IBM SPSS Statistics, Version 27 (IBM Corp., Armonk, NY). Continuous variables were expressed as median with interquartile range. Categorical variables were summarized as absolute and relative frequencies. Comparisons between categorical variables were conducted using the χ^2^ test (or Fisher’s exact test when expected cell counts were below five). As we did not assume normal distribution of continuous variables, the non-parametric Mann–Whitney *U* test was used, providing a conservative assessment of differences between groups even in the case of normal distribution. Survival analyses were carried out according to the Kaplan–Meier method, and intergroup comparisons of survival distributions were assessed using the log-rank (Mantel–Cox) test. To evaluate the prognostic significance of clinicopathological variables, we employed a multivariable Cox proportional hazards regression model utilizing a Bonferroni correction to account for multiple testing. For inter-reader agreement, overall agreement and weighted Cohen’s kappa were used. All statistical tests were two-sided, and a *P* value <0.05 was considered statistically significant.

### Patient identification and dropout

Initial screening using ICD-10 and OPS codes identified 242 patients. After manual review, four were excluded due to tumor location or histopathology. Additional exclusions included 16 cases without contrast-enhanced CT within 4 weeks of EUS, 5 with non-contrast CT, and 58 with only arterial-phase imaging. The final cohort therefore comprised 159 patients. Median follow-up in the cohort was 52.0 months.

## Results

### Node-RADS scoring

The final Node-RADS score was determined by the highest score at any location. In our cohort, 75, 18, 26, 18, and 22 patients had scores of 1-5, respectively. Inter-reader agreement was high, with an overall agreement of 92.2% and a Cohen’s kappa of 0.972. Of note, seven patients scored 5 solely due to lymph node size (‘5 through bulk’) without other malignant features. Kaplan–Meier analysis (Supplementary Figure S1 and Supplementary Table S1, available at https://doi.org/10.1016/j.esmogo.2026.100321) showed no significantly improved OS in Node-RADS 5 due to bulk compared with Node-RADS 5 from other features (median OS: Node-RADS 1: 68.8 months; Node-RADS 2: 45.3 months; Node-RADS 3: 42.6 months; Node-RADS 4: 49.1 months; Node-RADS 5: 51.9 months; Node-RADS 5 through bulk: 23.1 months; χ^2^ = 18.2, *P* = 0.003 for difference between groups). Deeper subgroup analysis was hindered by small group sizes in respective subgroups. We therefore decided to not distinguish between Node-RADS 5 through bulk and Node-RADS 5. Since Node-RADS is not validated in esophageal adenocarcinomas and survival was similar across Node-RADS 2-5, we dichotomized the cohort for further analysis: Node-RADS 1 as nodal negative (nN−) and Node-RADS 2-5 as nodal positive (nN+).

### Clinicopathological parameters

Following this definition, in our cohort, 75 patients were Node-RADS negative (nN−) and 84 patients Node-RADS positive (nN+). For an overview of clinical and pathological parameters in our cohort, as well as in the nN+ and nN− subgroups, see Table 1.

**Table 1:** Overview of overall clinicopathological parameters and in subgroups defined by their Node-RADS positivity

Parameter	Whole cohort (*n* = 159)	Node-RADS+ (*n* = 84)	Node-RADS− (*n* = 75)	*P* value
Age (years)	65.8 ± 15	66.0 ± 16	65.6 ± 16	0.684
Gender (male)	132 (83.0)	72 (85.7)	60 (83.3)	0.400
BMI (kg/m^2^)	26.5 ± 5.0	26.4 ± 5.9	26.5 ± 3.85	0.478
uT (≥uT3)	119 (74.8)	64 (76.2)	55 (73.3)	0.717
**uN (+)**	**112 (70.4)**	**70 (83.3)**	**42 (56.0)**	**0.001**
cM (+)	0	0	0	1.00
Grading				0.178
G1	7 (4.4)	2 (2.8)	5 (8.5)	—
G2	70 (44.0)	36 (50.7)	34 (57.6)	—
G3	53 (33.3)	33 (46.5)	20 (33.9)	—
**ypN (+)**	**74 (46.5)**	**50 (59.5)**	**24 (32.0)**	**0.001**
**ypT (**≥**ypT3)**	**77 (48.4)**	**47 (56.0)**	**30 (40.0)**	**0.049**
ypL (+)	44 (29.6)	27 (33.3)	17 (23.3)	0.168
ypV (+)	8 (5.1)	6 (7.2)	2 (2.7)	0.198
Surgical approach				0.786
Open	15 (9.4)	9 (10.6)	6 (8.0)	—
Laparoscopic	67 (42.1)	36 (42.9)	31 (41.3)	—
Hybrid	32 (20.1)	17 (20.2)	15 (20.0)	—
Robotic	44 (27.7)	21 (25.0)	23 (30.7)	—
ASA ≤3	89 (56.0)	41 (48.8)	48 (64.0)	0.054
Neoadjuvant treatment				0.607
Chemotherapy (FLOT/FLO)	143 (89.9)	77 (91.7)	66 (88.0)	—
Radiochemotherapy (CROSS)	16 (10.1)	7 (8.3)	9 (12.0)	—
Localization				0.623
Esophageal	58 (36.5)	29 (34.5)	29 (38.7)	—
AEG Siewert I	101 (63.5)	55 (65.5)	46 (61.3)	—

Categorical data are described as *n* (%). Continuous variables are shown as median and interquartile range. Statistical testing by Mann–Whitney *U* test for continuous variables and Fisher’s exact test for categorical data. Missing values: grading 29 patients; surgical approach 1. Statistical significant parameters (*P* < 0.05) are indicated in bold.

AEG, adenocarcinoma of the esophagogastric junction; ASA, American Society of Anesthesiologists score; BMI, body mass index; cM, clinical metastasis; CROSS, chemoradiotherapy regimen (carboplatin, paclitaxel + radiotherapy); EUS, endoscopic ultrasound; FLOT/FLO, perioperative chemotherapy regimen (5-fluorouracil, leucovorin, oxaliplatin ± docetaxel); Gx, tumor grading; Node-RADS, Node Reporting and Data System; uT/uN, EUS tumor stage and nodal stage; ypL, lymphatic invasion; ypN, pathological nodal stage; ypT, pathological tumor stage; ypV, venous invasion.

Pretherapeutic endosonographic staging (uN; see below) as well as histopathological nodal status (ypN; see below) and tumor stage (χ^2^ = 4.01, *P* = 0.045, ϕ = 0.16) following neoadjuvant treatment showed statistically significant differences between groups defined by Node-RADS positivity.

### Correlation of lymph node staging by EUS, ypN status, tumor regression grade, and Node-RADS

As a baseline, we investigated the correlation between histopathological nodal status after neoadjuvant treatment (ypN) and endosonography (uN). ypN and uN status showed a strong correlation (χ^2^ = 26.9, *P* < 0.001, ϕ = 0.41; double positive *n* = 67, uN+ ypN− *n* = 45, uN− ypN+ *n* = 7, double negative *n* = 40) This translated to a sensitivity of 90.5% (67/74), a specificity of 47.1% (40/85), or a positive predictive value (PPV) of 59.8% (67/112) and a negative predictive value (NPV) of 85.1% (40/47), with an overall accuracy of 67.3% (107/159).

We investigated the correlation between Node-RADS-based nodal status (nN) and endosonography. nN and uN status showed a strong correlation (χ^2^ = 20.3, *P* < 0.001, ϕ = 0.36; double positive *n* = 70, uN+ nN− *n* = 42, uN− nN+ *n* = 14, double negative *n* = 33), with most discordant cases being uN+ but nN− (75%, 42/56). This translated to a sensitivity of 62.5% (70/112), a specificity of 70.2% (33/47), or a PPV of 83.3% (70/84) and an NPV of 44.0% (33/75), with an overall accuracy of 64.8% (103/159) (Table 2).

**Table 2:** Contingency table for the correlation between nodal status by Node-RADS (nN) and endosonography (uN)

Correlation nN/uN	uN+	uN−	Total
nN+	70	14	84
nN−	42	33	75
Total	112	47	159

Node-RADS exhibited a sensitivity of 62.5% (70/112), a specificity of 70.2% (33/47), a positive predictive value of 83.3% (70/84), and a negative predictive value of 44.0% (33/75) for the prediction of nodal positivity by endosonography, with an overall accuracy of 64.8% (103/159). χ^2^ = 20.3, *P* < 0.001, ϕ = 0.36; true positive *n* = 70, false negative *n* = 42, false positive *n* = 14, true negative *n* = 33.

Node-RADS, Node Reporting and Data System.

**Figure 1:**
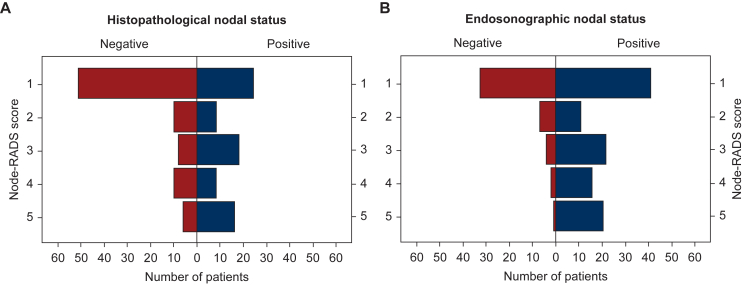
**Node-RADS score in patients staged nodal positive by histopathology or EUS.** (A) Correlation of Node-RADS and ypN status (χ^2^ = 20.3, *P* < 0.001, ϕ = 0.36). (B) Correlation of Node-RADS and uN status (χ^2^ = 17.9, *P* < 0.001, ϕ = 0.34). EUS, endoscopic ultrasound; Node-RADS, Node Reporting and Data System.

Pathological lymph node status after neoadjuvant treatment also correlated significantly with nN, though with a smaller effect size (χ^2^ = 17.9, *P* < 0.001, ϕ = 0.34; double positive *n* = 50, ypN+ nN− *n* = 24, ypN− nN+ *n* = 34, double negative *n* = 51) (Figure 1). This translated to a sensitivity of 67.6% (50/74), a specificity of 60.0% (51/85), a PPV of 59.5% (50/84), and an NPV of 68.0% (51/75), with an overall accuracy of 63.5% (101/159) (Table 3).

**Table 3:** Contingency table for the correlation between nodal status by Node-RADS (nN) and pathological nodal status after neoadjuvant treatment (ypN)

Correlation nN/ypN	ypN+	ypN-	Total
nN+	50	34	84
nN−	24	51	75
Total	74	85	159

Node-RADS exhibited a sensitivity of 67.6% (50/74), a specificity of 60.0% (51/85), a positive predictive value of 59.5% (50/84), and a negative predictive value of 68.0% (51/75) for the prediction of pathological nodal positivity (ypN+), with an overall accuracy of 63.5% (101/159). Of note, performance metrics are approximate and should be interpreted with caution, as nN reflects baseline status and nodal involvement may change (progress or regress) under neoadjuvant therapy. χ^2^ = 17.9, *P* < 0.001, ϕ = 0.34; true positive *n* = 50, false negative *n* = 24, false positive *n* = 34, true negative *n* = 51.

Node-RADS, Node Reporting and Data System.

Of note, when investigating sensitivity and specificity of uN and nN status with ypN status, the possibility for both regression and progression under neoadjuvant treatment should be kept in mind.

Node-RADS positivity was not correlated with treatment response to neoadjuvant treatment as assessed by Becker score (χ^2^ = 1.44, *P* = 0.696, ϕ = 0.095).

### Node-RADS positivity at diagnosis is predictive of worse survival

**Figure 2:**
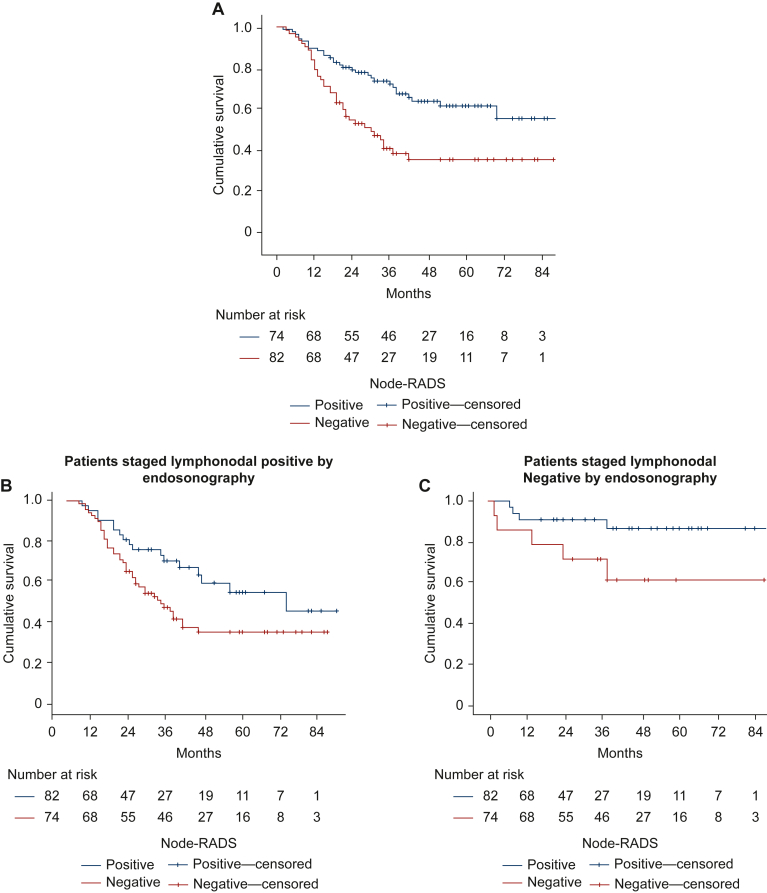
**Node-RADS positivity correlates with shorter overall survival in patients with adenocarcinoma of the esophagus.** (A) Patients classified as Node-RADS− have a significantly longer overall survival when compared with cases classified as Node-RADS+ (65.4 versus 46.2 months, χ^2^ = 10.6, *P* = 0.001). (B, C) This association remained significant after stratification by endosonographic nodal status, both in uN− patients (77.0 versus 65.3 months) and in uN+ patients (55.2 versus 42.4 months); Mantel–Cox pairwise over strata, χ^2^ = 4.241, *P* = 0.039. Node-RADS, Node Reporting and Data System.

We next analyzed the correlation between Node-RADS positivity and OS. Patients with nN+ at diagnosis had significantly shorter OS than nN− patients (46.2 versus 65.4 months, χ^2^ = 12.85, *P* < 0.001; Figure 2). This effect remained significant in uN+ cases (42.6 versus 57.6 months, χ^2^ = 4.581, *P* = 0.032) and showed a trend in uN− cases (60.8 versus 81.2 months, χ^2^ = 3.589, *P* = 0.058; Figure 2B and C).

This strong correlation remained statistically significant when carrying out a multivariable analysis including age, sex, uN, uT stage, and American Society of Anesthesiologists physical status classification, with only nN and uN status remaining independently significant predictors [*P* = 0.007, Exp(B) = 2.101 and *P* = 0.004, Exp(B) = 3.041; see Table 4]. Both variables remained statistically significant after Bonferroni correction (nN *P*adj = 0.042 and uN *P*adj = 0.024).

**Table 4:** Multivariant Cox regression controlled for age, sex, uN and uT stage, and ASA classification

Variable	B	SE	Wald	*df*	*P* value	Exp(B)
**Node-RADS (nN+)**	**0.742**	**0.277**	**7.182**	**1**	**0.007**	**2.101**
Age (per year)	0.018	0.013	1.851	1	0.174	1.018
Sex (male)	0.256	0.347	0.546	1	0.46	0.774
uT (≥uT3)	0.363	0.292	1.541	1	0.214	1.437
**Endosonography (uN+)**	**1.112**	**0.383**	**8.44**	**1**	**0.004**	**3.041**
ASA (≥ASA 3)	0.387	0.259	2.232	1	0.135	1.473

ASA, American Society of Anesthesiologists physical status classification; B, regression coefficient; *df*, degrees of freedom; Exp(B), hazard ratio; Node-RADS, Node Reporting and Data System; SE, standard error; uN, endoscopic ultrasound-based nodal status; uT, endoscopic ultrasound-based tumor stage.

### Discordant staging findings exhibit intermediate OS rate compared with double-positive or double-negative cases

**Figure 3:**
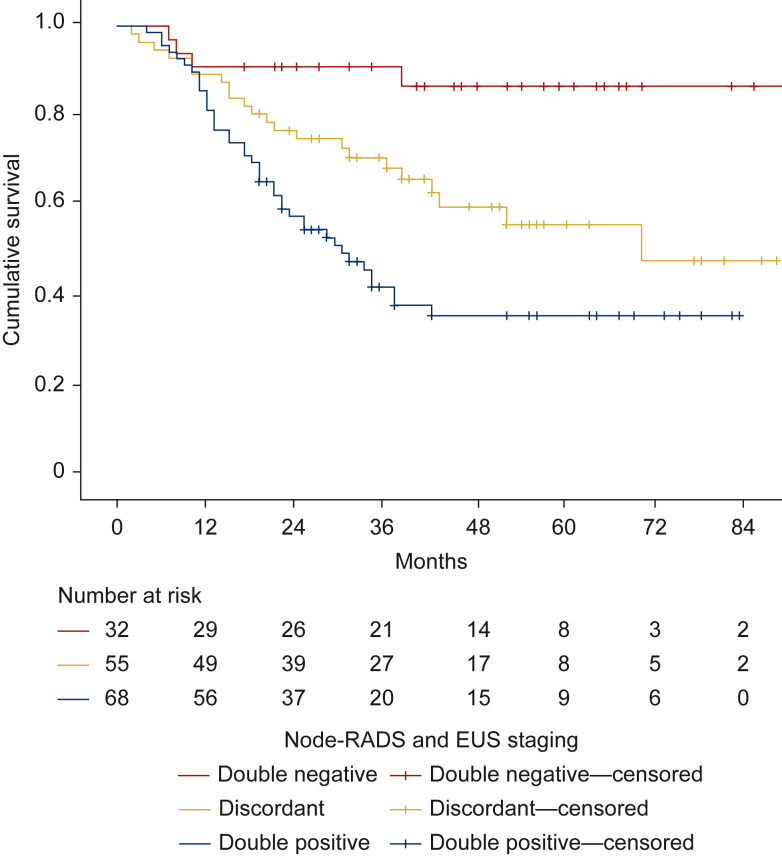
**Discordant staging by Node-RADS and EUS identifies patients with intermediate survival.** Overall survival node staging at diagnosis double negative nN−/uN−, discordant nN−/uN+ or nN+/uN−, and double positive uN+/nN+. Patients with double positive exhibit longest overall survival (81.2 months) when compared with discordant (58.6 months) or double-negative patients (42.6 months) (double negative versus discordant *P* = 0.011, χ^2^ =6.525; double negative versus double positive *P* < 0.001, χ^2^ =15.498; discordant versus double positive χ^2^ = 5.696, *P* = 0.017). EUS, endoscopic ultrasound; Node-RADS, Node Reporting and Data System.

We further analyzed OS by combined nodal status: double-negative (nN−/uN−), double-positive (nN+/uN+), and discordant cases (nN and uN differing). OS differed significantly across groups, with discordant cases showing intermediate survival (58.6 months) compared with double-negative (81.2 months) and double-positive (42.6 months) cases (Figure 3). Pairwise comparisons demonstrated significant differences between double-negative and discordant cases: double negative versus discordant, *P* = 0.011, χ^2^ = 6.525; double negative versus double positive, *P* < 0.001, χ^2^ = 15.498; discordant versus double positive, *P* = 0.017, χ^2^ = 5.696.

## Discussion

In this retrospective study, we compared CT-based lymph node staging using Node-RADS with EUS in patients with locally advanced esophageal adenocarcinomas undergoing curative-intent multimodal therapy.

The Node-RADS system scores lymph nodes from 1 to 5, with higher scores indicating a greater likelihood of malignancy. In our cohort, inter-reader agreement was excellent with a weighted Cohen’s kappa of 0.973; this agreement is most probably high due to two very experienced radiologists, both well trained in Node-RADS, and the monocentric nature of our study. Further multicenter studies with differently experienced radiologists are needed before broad implementation. While useful for radiologic classification, clinical decisions often require dichotomization into lymph node positive and node negative. Leonhardi et al. suggested a Node-RADS cut-off of ≥3 for predicting histopathological nodal positivity in esophageal cancer,^13^ though interpretation is limited as neoadjuvant treatment is not taken into account, indicating that the optimal clinical threshold remains unclear. In gastric cancer, Node-RADS was shown to improve diagnostic accuracy over CT-based staging by size,^16^^,^^17^ and was shown to be predictive of residual lymph node disease after neoadjuvant treatment with prognostic impact on OS.^18^

In our cohort, Node-RADS demonstrated high concordance with EUS-based staging and correlated with histopathological nodal positivity after neoadjuvant therapy. Node-RADS positivity at diagnosis was also a strong predictor of worse OS. Notably, different Node-RADS scores did not clearly stratify survival, with score 2 showing similar outcomes to score 5. Accordingly, we defined Node-RADS ≥2 as positive in this study. However, without a definitive ‘ground truth’ for nodal involvement, survival-based approximation should not be interpreted as a ‘true’ PPV. Further studies are required to establish optimal cut-offs for esophageal adenocarcinomas.

We observed high concordance between EUS and Node-RADS nodal staging. Node-RADS positivity correlated with histopathological nodal involvement after neoadjuvant therapy and with poorer OS. Patients with discordant results had intermediate outcomes, most commonly showing positive EUS but negative Node-RADS nodal status. Despite EUS being the current standard of care for nodal assessment,^2^ up to 50% of cT2N0-staged gastric and esophagogastric junction cancers are understaged versus pathology,^19^ highlighting sensitivity limitations. EUS-based staging exhibited a high sensitivity of 90.5% and a low specificity of 47.1% for histopathological nodal positivity after neoadjuvant treatment. This sensitivity is in line with prior reports^8^ in esophageal cancer, and the rather low specificity can partly be explained by regression of a subgroup of patients over the course of neoadjuvant treatment. In our predominantly nodal-positive cohort, discordant cases were mostly uN+/nN−, suggesting a possible tendency of EUS toward overstaging as previously reported in literature.^8^^,^^20^ Importantly, no widely accepted standardized criteria exist for EUS-based definition of nodal involvement in esophageal adenocarcinoma,^21^ though features such as nodes ≥10 mm, round shape, sharp borders, and absence of central vessels are commonly used.^22^ A data-driven standardization effort, similar to Node-RADS, could reduce intra- and interobserver variability in EUS nodal staging and is warranted.

Clinically, accurate staging is crucial because over- and understaging carry different risks. Multimodal therapy significantly improves survival in locally advanced disease, as shown in the MAGIC trial^23^ and with modern protocols like FLOT.^4^ Understaging patients who could benefit from such therapy may be more harmful than overtreatment, given the substantial survival benefit. Nonetheless, therapy de-escalation should be applied whenever feasible.

Our study suggests that combining Node-RADS with EUS provides additional information beyond EUS alone, the current ‘gold standard’ for nodal staging. More broadly, the integration of all available patient data—including radiologic, clinical, and molecular information—could improve individualized risk assessment and enable better tailored treatment plans. Further studies are needed to explore this approach.

Several limitations of our study should be acknowledged. Many patients were excluded due to inadequate CT contrast phases, limiting the generalizability of our finding to all cases. Optimal assessment would require histopathological nodal assessment in the absence of neoadjuvant therapy, as nodal status may change with treatment, preventing definitive determination of Node-RADS sensitivity and specificity in this retrospective, multimodal setting. Reported PPVs and NPVs should be interpreted with caution. Comparisons with EUS and survival outcomes therefore provide the best available approximation. This study did not investigate changes of Node-RADS under neoadjuvant treatment; this temporal component is of high interest and might give additional clinically useful information. Finally, the retrospective and single-center design further limits the generalizability of our findings.

In summary, CT-based nodal staging using the Node-RADS framework showed strong agreement with EUS, correlated with pathological nodal status after neoadjuvant treatment, and was independently associated with shorter OS. These results support a potential role for Node-RADS in preoperative staging, but prospective, multicenter studies are needed to define optimal cut-offs and clarify the clinical value of multidimensional lymph node staging in esophageal adenocarcinomas.

## Disclosure

JJS received advisory fees from Takeda unrelated to the subject of this study. BS received consulting and speaker fees from Abbvie, Abivax, AlfaSigma, Boehringer Ingelheim, Bristol Myers Squibb, CED Service GmbH, Dr. Falk Pharma, Endpoint Health, Eli Lilly, Galapagos, Janssen/Johnson & Johnson, Materia Prima, MD Education, MSD, Pfizer, Takeda, Tr1xBio, and Wedbush Securities and grant money from Pfizer, all unrelated to this topic. All other authors have declared no conflicts of interest.
