# Supplementary material for: Additional benefit of standardized computed tomography-based lymph node assessment utilizing Node-RADS in esophageal adenocarcinoma
Source: ESMO Gastrointest Oncol. 2026 Mar 26;12:100321. doi: 10.1016/j.esmogo.2026.100321 (PMC13059021; doi:10.1016/j.esmogo.2026.100321)
Supplement: Supplementary Table S1 [file mmc1.docx]

**Supplement**

**Supplementary table:**

| Parameter | Whole cohort (n=159) | Node-RADS cut off 3  (n=66) | Node-RADS cut off 2  (n=93) | p-value |
| --- | --- | --- | --- | --- |
| Age (years) | 65.6 ± 15 | 66.0 ± 15 | 65.0 ± 18.0 | 0.189 |
| Gender (male) | 132 (83.0%) | 56 (84.8%) | 76.0 (81.7%) | 0.605 |
| BMI (kg/m2) | 26.0 ± 4.97 | 26.2 ± 5.5 | 25.8 ± 4.6 | 0.478 |
| uT (> T3) | 119 (74.8%) | \| 51 (77.3%) \| \| --- \| | 68 (73.1%) | 0.552 |
| uN (+) | 112 (70.4%) | 59 (89.4%) | \| 53 (57.0%) \| \| --- \| | **0.001** |
| cM (+) | 0 | 0 | 0 | 1.00 |
| Grading  G1  G2  G3 | 7 (4.4%)  70 (44.0%)  53 (33.3%) | 2 (3.0%)  25 (37.9%)  30 (45.5%) | 5 (5.4%)   \| 45 (48.4%) \| \| --- \| \| 23 (24.7%) \| | **0.049** |
| Surgical approach  Open  Laparoscopic  Hybrid  robotic | \| 15 (9.4%) \| \| --- \|   67 (42.1%)   \| 32 (20.1%) \| \| --- \| \| 44 (27.7%) \| | 7 (10.6%)   \| 30 (45.5%) \| \| --- \| \| 13 (19.7%) \| \| 15 (22.7%) \| | \| 8 (8.6%) \| \| --- \| \| 37 (39.8%) \| \| \| 19 (20.4%) \| \| \| 29 (31.2%) \| \| | 0.691 |
| ASA ≤3 | 89 (56.0%) | 31 (47.0%) | 58 (62.4%) | 0.074 |
| Neoadjuvant treatment Chemotherapy (FLOT/FLO)  Radiochemotherapy (CROSS) | 143 (89.9%)  16 (10.1%) | \| 59 (89.4%) \| \| --- \|   7 (10.6%) | \| 84 (90.3%) \| \| \| --- \| --- \| \| 9 (9.7%) \| | 0.368 |
| Localization  Esophageal  AEG Siewert I | \| 58 (36.5%) \| \| --- \| \| 101 (63.5%) \| \| | \| 23 (34.8%) \| \| --- \| \| 43 (65.2%) \| | \| 35 (37.6%) \| \| --- \| \| 58 (62.4%) \| | 0.741 |

**Supplementary Table 1**: Clinicopathologic parameters when a cut-off of 3 (as defined in the initial publication) was used for definition of Node-RADS positivity. Abbreviations: Node-RADS, Node Reporting and Data System. BMI, body mass index; uT/uN, EUS-based tumor/nodal stage; cM, clinical metastasis; Gx, tumor grade; ASA, American Society of Anesthesiologists score; FLOT/FLO, perioperative chemotherapy regime; CROSS, chemoradiotherapy regimen (Carboplatin, Paclitaxel + Radiotherapy); AEG, adenocarcinoma of the esophagogastric junction;

**Supplementary Figure**


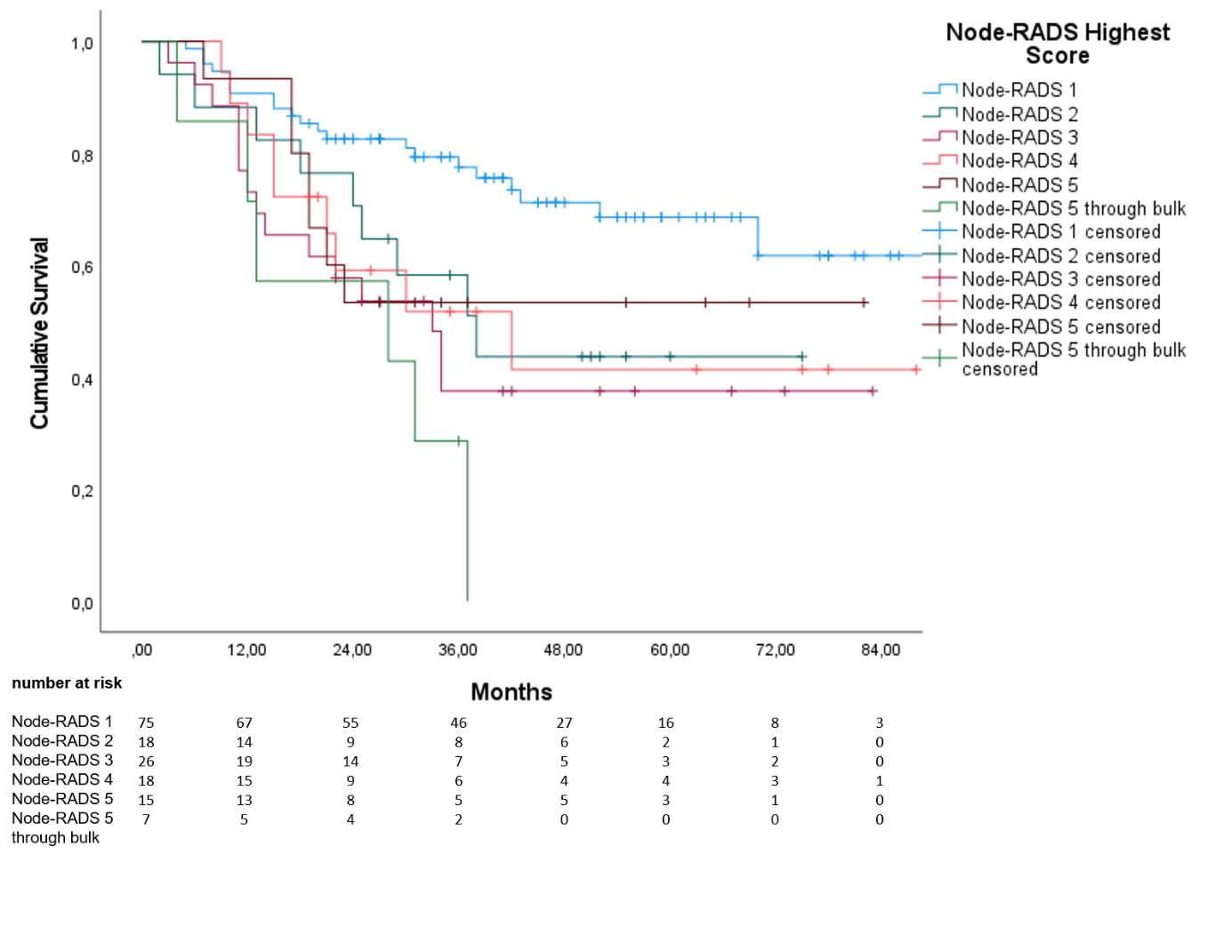


**Supplementary Figure 1**: Kaplan Meier plots for overall survival stratified by groups defined by Node-RADS status. Patients scored as Node-RADS 5 because of a lymph node bulk do not display a improved survival compared to patients with Node-RADS positivity due to other factors (Node-RADS 1: 68.8 months; Node-RADS 2: 45.3 months; Node-RADS 3: 42.6 months; Node-RADS 4: 49.1 months; Node-RADS 5: 51.9 months; Node-RADS 5 through bulk: 23.14 months. **χ²** 18,2 p=0.003 for difference between groups.
