# Supplementary figures and images for: Additional benefit of standardized computed tomography-based lymph node assessment utilizing Node-RADS in esophageal adenocarcinoma
Source: ESMO Gastrointest Oncol. 2026 Mar 26;12:100321. doi: 10.1016/j.esmogo.2026.100321 (PMC13059021; doi:10.1016/j.esmogo.2026.100321)

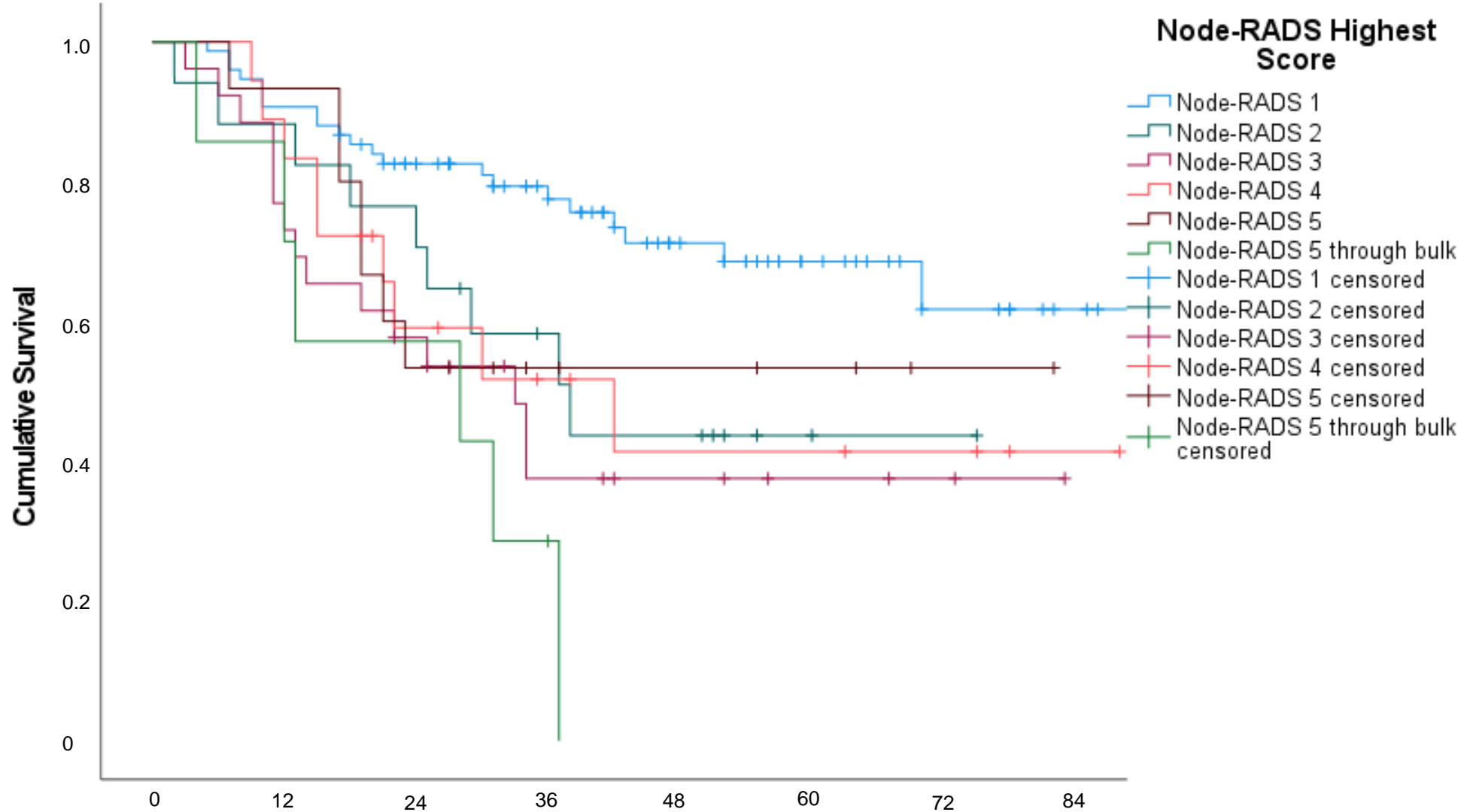

number at risk

Months

|                          |    |    |    |    |    |    |   |   |
|--------------------------|----|----|----|----|----|----|---|---|
| Node-RADS 1              | 74 | 67 | 55 | 46 | 27 | 16 | 8 | 3 |
| Node-RADS 2              | 16 | 14 | 9  | 8  | 6  | 2  | 1 | 0 |
| Node-RADS 3              | 25 | 19 | 14 | 7  | 5  | 3  | 2 | 0 |
| Node-RADS 4              | 17 | 15 | 9  | 6  | 4  | 4  | 3 | 1 |
| Node-RADS 5              | 14 | 13 | 8  | 5  | 5  | 3  | 1 | 0 |
| Node-RADS 5 through bulk | 6  | 5  | 4  | 2  | 0  | 0  | 0 | 0 |

Supplement: Supplementary Figure S1 [file mmc2.pdf]
